# No Autopsies on COVID-19 Deaths: A Missed Opportunity and the Lockdown of Science

**DOI:** 10.3390/jcm9051472

**Published:** 2020-05-14

**Authors:** Monica Salerno, Francesco Sessa, Amalia Piscopo, Angelo Montana, Marco Torrisi, Federico Patanè, Paolo Murabito, Giovanni Li Volti, Cristoforo Pomara

**Affiliations:** 1 Department of Medical, Surgical and Advanced Technologies “G.F. Ingrassia”, University of Catania, 95121 Catania, Italy; monica.salerno@unict.it (M.S.); angelomontana49@gmail.com (A.M.); marco.torrisi.1992@gmail.com (M.T.); federicopatane90@gmail.com (F.P.); 2 Department of Clinical and Experimental Medicine, University of Foggia, 71122 Foggia, Italy; francesco.sessa@unifg.it; 3 Department of Law, Forensic Medicine, Magna Graecia University of Catanzaro, 88100 Catanzaro, Italy; amaliapiscopo@gmail.com; 4 Department of General surgery and medical-surgical specialties, University of Catania, 95121 Catania, Italy; paolo.murabito@unict.it; 5 Department of Biomedical and Biotechnological Sciences, University of Catania, 95121 Catania, Italy

**Keywords:** COVID-19, infectious diseases, autopsy, diagnosis

## Abstract

Background: The current outbreak of COVID-19 infection, which started in Wuhan, Hubei province, China, in December 2019, is an ongoing challenge and a significant threat to public health requiring surveillance, prompt diagnosis, and research efforts to understand a new, emergent, and unknown pathogen and to develop effective therapies. Despite the increasing number of published studies on COVID-19, in all the examined studies the lack of a well-defined pathophysiology of death among patients who died following COVID-19 infection is evident. Autopsy should be considered mandatory to define the exact cause of death, thus providing useful clinical and epidemiologic information as well as pathophysiological insights to further provide therapeutic tools. Methods: A literature review was performed on PubMed database, using the key terms: “COVID-19”, “nCov 19”, and “Sars Cov 2”. 9709 articles were retrieved; by excluding all duplicated articles, additional criteria were then applied: articles or abstracts in English and articles containing one of the following words: “death”, “died”, “comorbidity”, “cause of death”, “biopsy”, “autopsy”, or “pathological”. Results: A total of 50 articles met the inclusion criteria. However, only 7 of these studies reported autopsy-based data. Discussion: The analysis of the main data from the selected studies concerns the complete analysis of 12,954 patients, of whom 2269 died (with a mortality rate of 17.52%). Laboratory confirmation of COVID-19 infection was obtained in all cases and comorbidities were fully reported in 46 studies. The most common comorbidities were: cardiovascular diseases (hypertension and coronary artery disease), metabolic disorders (diabetes, overweight, or obesity), respiratory disorders (chronic obstructive pulmonary disease), and cancer. The most common reported complications were: acute respiratory distress syndrome (ARDS), acute kidney injury, cardiac injury, liver insufficiency, and septic shock. Only 7 papers reported histological investigations. Nevertheless, only two complete autopsies are described and the cause of death was listed as COVID-19 in only one of them. The lack of postmortem investigation did not allow a definition of the exact cause of death to determine the pathways of this infection. Based on the few histopathological findings reported in the analyzed studies, it seems to be a clear alteration of the coagulation system: frequently prothrombotic activity with consequent thromboembolism was described in COVID-19 patients. As a scientific community, we are called on to face this global threat, and to defeat it with all the available tools necessary. Despite the improvement and reinforcement of any method of study in every field of medicine and science, encouraging the autopsy practice as a tool of investigation could also therefore, help physicians to define an effective treatment to reduce mortality.

## 1. Background

The current outbreak of COVID-19 infection, which started in Wuhan, Hubei province, China, in December 2019 is an ongoing challenge and a significant threat to public health requiring surveillance, prompt diagnosis, research efforts to understand a new, emergent, and unknown pathogen and to develop effective interventions [1,2,3].

Despite the increasing number of published studies on COVID-19, there is an evident lack, in all the examined studies, of a well-defined pathophysiology of death among patients who died following COVID-19 infection.

The loss of potential key information about the real mechanisms underlying death due to COVID-19 infections does not allow a real evaluation of COVID-19 mortality, which could even be overestimated given that the precise cause of death remains elusive.

The autopsy should be considered mandatory in order to define the exact cause of death, thus providing useful clinical and epidemiologic information. The role of autopsy still remains unchanged despite the decline in autopsy rates. During the last few decades, several factors such as time constraints, attitudes (of clinicians, pathologists, families, or administrators), and costs (professional and overheads) have negatively influenced the autopsy rate, leading to the lack of substantial information [4]. In 1996, Schwartz and Herman [5] highlighted the importance of autopsy, particularly in emerging and re-emerging infectious diseases.

During the recent outbreak of the Middle East respiratory syndrome (MERS), the value of this investigation tool was emphasized. The first autopsy report was published in 2016, two years after it had been performed in 2014, while the infection had started in 2012. Analyzing the data reported by Ng et al. [6], the autopsy provided unprecedented, clinically-relevant insights about how this unknown infection had progressed, challenging previously accepted ideas about MERS. Autopsy provides critical information regarding emerging or unknown infectious diseases [5,7].

## 2. Methods

### 2.1. Database Search Terms and Timeline

We performed a literature search on online resources (PubMed database) from 1 January 2019 to 28 April 2020, using the following key search terms: “COVID-19”, “nCov 19”, and “Sars Cov 2”.

### 2.2. Study Selection

**Figure 1:**
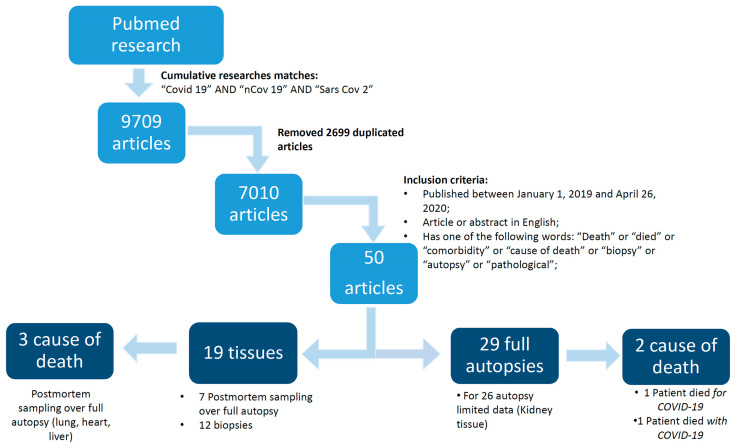
The search strategy used for literature review.

We retrieved 9709 articles; by excluding all duplicated articles, additional criteria were then applied: articles or abstract in English and articles containing one of the following words: ““death”, “died”, “comorbidity”, “cause of death”, “biopsy”, “autopsy”, or “pathological”. To identify further studies that met the inclusion criteria, the references of the selected articles were also reviewed. Articles were excluded if they did not meet the inclusion criteria outlined previously. Figure 1 summarizes the data obtained after our PubMed literature search.

## 3. Results

A total of 50 articles met the inclusion criteria out of the 7010 articles found [8,9,10,11,12,13,14,15,16,17]. The main characteristics of each selected article are summarized in Table 1.

Analyzing Table 1, only in seven papers had the analysis of tissue samples been performed.

Tian et al. [9] analyzed two patients infected by COVID-19 with lung cancer. Two lung biopsies were performed before COVID-19 diagnosis and showed the following: alveolar edema and proteinaceous exudates, vascular congestion but patchy and mild inflammatory infiltration, focally fibrin clusters mixed with mononuclear inflammatory cells, and multinucleated giant cells were noted in the airspaces; no significant neutrophil infiltration was present in lung tissue, pneumocyte hyperplasia and interstitial thickening were also observed, indicating an ongoing reparative process. Suspected viral inclusions were also noted in some of these cells. At the time of manuscript preparation (submission on the 18 of February 2020), they have remarked that no full autopsy was performed on patients with COVID-19. At the same time, no data on lung biopsies for the COVID-19 infection were collected. Moreover, the authors highlighted the importance of the autopsy specimens to perform routine histopathological and immunohistochemical analyses combined with other investigations, such as RT-PCR, to understand better the mechanism of action of SARS-CoV-2 [9].

Respiratory tract involvement is further described by Xu et al. [11], who described for the first time the pathological findings of COVID-19 associated with acute respiratory distress syndrome. The authors collected post-mortem specimens from lung, heart, and liver tissues. Specimens from lungs showed the following: desquamation of pneumocytes and hyaline membrane formation, indicating acute respiratory distress syndrome, with interstitial mononuclear inflammatory infiltrates, dominated by lymphocytes. Pneumocytes were characterized by viral cytopathic-like changes. In their conclusions, the authors highlighted that their clinical and pathological findings in the reported case of COVID-19 were not only useful to identify the cause of death, but also to provide new insights into the pathogenesis of SARS-CoV-2-related pneumonia. Moreover, these findings could be beneficial for physicians to formulate a suitable therapeutic strategy for similar severe patients, reducing mortality.

Barton et al. [38] described the findings of the two complete autopsies performed on COVID-19 patients. It is important to note that even if the macroscopic investigation was performed on several organs, the histological and immunohistochemical examinations were carried out on lung specimens. In the first case, both microscopic investigations reported the presence of thrombi within a few small pulmonary artery branches. Congestion of alveolar septal capillaries and edema fluid within the airspaces were also reported. For these reasons, the cause of death was listed as COVID-19. Conversely, in the second case there was no evidence of diffuse alveolar damage (DAD), even if immunohistochemistry showed similar findings to the other case. Therefore, considering the presence of other comorbidities, the cause of death was listed as “complications of hepatic cirrhosis”, listing the COVID-19 infection under other significant conditions.

Magro et al. [47] examined lung and cutaneous tissues sampled from five patients with COVID-19 infection and severe respiratory failure, three of whom also had features consistent with a systemic procoagulant state. They performed histologic and immunohistochemical investigations, defining a pattern of cutaneous and pulmonary pathology involving microvascular injury and thrombosis.

Cai et al. [51] reported seven biopsies collected from COVID-19 patients during the perioperative period of lung resection. The histopathological investigation was performed to obtain information about the lung tumor. Interstitial inflammation with plasma cell and macrophage infiltration was detected.

Karami et al. [52] described the first case of pregnancy mortality due to COVID-19. A partial autopsy was performed; indeed, the authors reported that the patient underwent lung autopsy. The histologic investigation highlighted alveolar spaces with focal hyaline membrane, pneumocyte proliferation, and metaplastic changes. Moreover, many inflammatory cells were found. Nevertheless, the authors reported that these findings were not enough to define the exact cause of death. Moreover, they said that these difficulties can be related to the absence of published evidence on COVID-19, especially in pregnant patients.

Tian et al. [53] reported the postmortem investigations of core biopsies of lung, liver, and heart in four patients who died of COVID-19 pneumonia. Even if several important findings were described, the absence of complete autopsies did not provide all the necessary information.

Su et al. [55] focused their study on the autopsy renal tissue sampled in patients who died of COVID-19, reporting direct adverse effects of the virus on this tissue.

No evidence of biopsies or autopsy samples were described in the other selected papers.

In their different studies, Guan [10] and Peng [8] focused their analysis on the outcome of different cohorts of COVID-19 patients. To evaluate the outcome of infected patients, Guan et al. analyzed the medical records and compiled data for hospitalized patients and outpatients with laboratory-confirmed COVID-19 infection, reporting a mortality of 1.4%.

Similarly, Peng et al. [8] analyzed the outcome of 112 COVID-19 patients affected by cardiovascular diseases. The authors reported that an overweight condition (BMI > 25 kg/m^2^) was significantly higher in critical or not-survivor patients compared to survivors.

Other retrospective studies were discussed. Clinical data and therapeutic options were analyzed by Kui et al. [12] performing a retrospective study analyzing the clinical data from 137 COVID-19 infected patients. The authors identified significant clinical characteristics and corresponding treatment principles for the disease.

Another retrospective study was performed to define the main characteristics of this infection. In the 17 cases analyzed, Wang et al. [13] described that the first deaths were mainly among older people with comorbidities or a history of surgery before admission.

Chen et al. reported the retrospective analysis of 99 patients positive for COVID-19 infection. Interestingly, 51% of patients suffered from chronic diseases, including endocrine system diseases, cardiovascular and cerebrovascular diseases, digestive system diseases, malignant tumors, respiratory system diseases, and nervous system diseases [17].

In another important retrospective study, the authors concluded that during the COVID-19 infection, both pulmonary and systemic inflammation can lead to multi-organ dysfunction in patients at high risk [23]. In their retrospective analysis, Deng et al. [25] reported that more patients who died showed characteristics of advanced age and pre-existing comorbidities.

Lescure et al. [30] described the clinical history of five COVID-19 patients discussing the different outcomes of the infection.

All the other contributions focused their attention on different characteristics of the comorbidities related to patients hospitalized with COVID-19 infection.

Analyzing the report about the mortality cases of COVID-19 in the Republic of Korea, Yuan et al. [20] described the mortality rate as being significantly higher in the COVID-19 patients with several comorbidities such as hypertension, diabetes, and cardiac disease.

Li et al. [22] reported that COVID-19 is associated with poor prognosis for patients undergoing thoracic operations, especially for those with chronic obstructive pulmonary disease (COPD). Several studies were performed analyzing clinical, laboratory, imaging features, and outcomes of COVID-19 confirmed cases reporting the idea that the presence of comorbidities is an important factor in the mortality rate of the COVID-19 patients [26,27,28,29,31,32,36,37,39,40,43,44,45,46,48,49]. Yang et al. [50] described a retrospective study conducted on deceased patients, reporting the presence of ARDS, myocardial injury, liver injury, renal insufficiency, and multiple organ dysfunction syndrome (MODS) in COVID-19 patients. Du et al. [42] identified four risk factors: age ≥65 years, preexisting concurrent cardiovascular or cerebrovascular diseases, CD3^+^CD8^+^ T cells ≤ 75 cell/μL, and cardiac troponin I ≥ 0.05 ng/mL; these last two factors were predictors for mortality of COVID-19 pneumonia patients. Moreover, other studies highlighted that males have a higher risk of death compared to females [34,35]. Furthermore, Li et al. [35] reported the importance of identifying critical patients quickly to improve their outcome. As described by Cheng et al. [41], acute kidney injury can be considered another important factor in patients’ outcome.

Huang C. et al. [15] analyzed the data of 41 patients admitted to hospital with laboratory-confirmed COVID-19 infection. About 33% of all patients had underlying diseases, such as diabetes, hypertension, and cardiovascular disease. Moreover, comparing the data of ICU patients with patients not treated in the ICU (102), they were older and with comorbidities.

The possible relationship between age, comorbidities, severity of respiratory distress, and death was then investigated by Yang et al. [14], who performed a full analysis of the electronic clinical medical records. Analyzing several aspects of deceased patients, they concluded that they were significantly older compared to survivors and with comorbidities and acute respiratory distress syndrome (ARDS).

Edrada et al. [56] reported two cases of COVID-19 patients; despite both patients being young adults with no significant past medical history, they had very different clinical courses. While one patient died after a few days of hospitalization, the other patient developed severe pneumonia and died. The different outcome is justified by the presence of several co-infections such as Influenza B, and Streptococcus pneumonia.

Zhang et al. [18] reported the existence of hypercoagulation status in critical COVID-19 patients, suggesting an anticoagulation therapy in similar patients. Fabre et al. [54] remarked that acute pulmonary embolism represents an important consequence in COVID-19 patients. The authors reported the case of a young woman presenting with severe pulmonary embolism, without any associated symptoms of infections, who died after 10 days of hospitalization. As reported by Guo et al. [21], myocardial injury represents an important factor associated with fatal outcomes of COVID-19. In the same way, Zhou et al. [24] reported that in COVID-19 patients, the presence of d-dimer levels greater than 1 μg/mL could help clinicians to identify patients with poor prognosis at an early stage. The higher d-dimer levels were also described by Bobin et al. [33]. As previously described, Magro et al. [47] tried to explain the pathogenesis of severe COVID-19 infection, describing an association with microvascular injury and thrombosis. Finally, in another recent report, d-dimer higher than 2.0 µg/mL on admission can be used as a predictor factor of in-hospital mortality in patients with COVID-19. Based on their data, d-dimer levels could be an early and helpful marker to improve the management of COVID-19 patients [57].

## 4. Discussion

**Figure 2:**
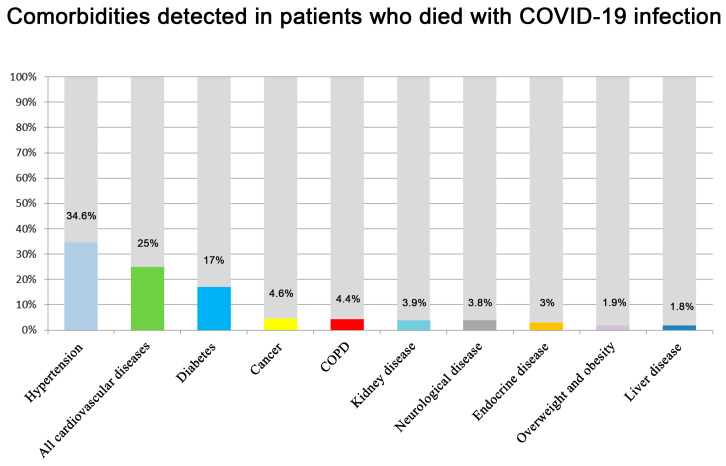
The histogram summarizes the comorbidity data in patients who died with COVID-19. Data are indicated in percentage for each comorbidity. Notably, percentage was obtained by indicating as the numerator the number of patients died who were positive for COVID-19 and affected by the specific comorbidity and, as the denominator, the total amount of patients who died and were positive for COVID-19.

The analysis of the main data from the selected studies concerns the complete analysis of 12,954 patients, of whom 2269 died (with a mortality rate of 17.52%). The weighted mean age was 54.88 years. These data are strongly influenced by the selection criteria of the present study: indeed, retrospective studies are frequently performed in hospitalized patients. Laboratory confirmation of COVID-19 infection was obtained in all cases. Comorbidities were fully reported in 46 studies, and are summarized in Figure 2. The most common comorbidities were cardiovascular diseases (hypertension and coronary artery disease), metabolic disorders (diabetes, overweight, or obesity), respiratory disorders (chronic obstructive pulmonary disease), and cancer. Complications were reported in 30 articles. The most common reported complications were: acute respiratory distress syndrome (ARDS), acute kidney injury, cardiac injury, liver insufficiency, and septic shock.

Fifty articles were included in the present review, and only seven papers reported histological investigations. Nevertheless, only Barton et al. [38] described two complete autopsies. In the autopsy report of the first case, the cause of death was listed as COVID-19 in a patient with coronary artery disease, indicated under “other contributing factors”. In the second case, the cause of death was listed as “complications of hepatic cirrhosis,” with muscular dystrophy, aspiration pneumonia, and COVID-19 listed as other significant conditions.

In the light of this literature review, the lack of postmortem investigation did not allow a definition of the exact cause of death. In this way, it is very complex to define the exact pathways of this infection. Based on the few histopathological findings reported in the analyzed studies, it may be thought that this virus exerts adverse effects on the lung and kidney. There is scarce evidence about heart and brain tissues. Moreover, it seems to be a clear alteration of the coagulation system; prothrombotic activity with consequent thromboembolism was frequently described in COVID-19 patients.

How can we estimate the real cause-specific mortality, including death rate, associated with COVID-19 when the cause of death is missing from the studies? How can we identify the additional care required for specific patient categories if there is still a lack of information about the real cause of death?

Such unsolved questions need to be addressed at the moment and should represent one of the goals of the scientific community.

Understanding the organ injury caused by COVID-19 and the underlying mechanisms could be a very helpful tool to optimize clinical management, helping clinicians to define a timely and effective treatment to reduce future mortality [58]. Indeed, after only about 1 month preliminary autopsy data (papers available in preprint version) suggested the importance of enhancing the host immune response against RNA viral infections. Recent reports about the pathological pathway of COVID-19 suggested that one of the most important consequences of this infection is the cytokine storm syndrome [15] that could be strictly linked with coagulopathy, generating acute pulmonary embolism caused by in-situ thrombosis [59,60]. In this way, a significant number of clinical trials are underway to define a useful therapy to attenuate cytokine storms [61].

For these reasons, it is important to also raise a political issue; although the World Health Organization suggested performing post-mortem examinations for those people who died with COVID-19 following recommended safety procedures [62], many Governments—including Italy—did not provide adequate tools to perform a sufficient number of autopsies. In Italy, despite the great action of our Government to face the epidemic, the Ministry of Health with a specific act (Circular of General Direction of Health Prevention) discourages the use of autopsy practice in COVID-19 deaths [63] and we are experiencing the incredible situation of having, unfortunately, thousands of deaths but almost no autopsies!

Considering that the COVID-19 infection is totally new, health care organizations and researchers have been publishing guidelines and recommendations to help health care providers proceed safely with various aspects of disease management and investigation [64,65]. Following these indications, it is possible to perform autopsies—that should be the way.

As recently argued by De Cock and Colleagues in the New England Journal of Medicine, despite the decline in autopsy rate a complete diagnostic autopsy remains the gold standard to determine why and how death happens, providing useful clinical information [66].

It is a fact that during the West Africa Ebola epidemic, in the Ebola virus disease (EVD) surveillance strategy, the RNA virus was isolated in body fluids days or months after the onset of the disease from any living or deceased individual who had, or had had, clinical symptoms compatible with EVD. Thanks to this procedure, it was possible to monitor the number of infected patients in order to recognize new sources of transmission and to control the epidemic phenomenon [67,68,69,70,71,72,73].

An equally effective method during yellow fever epidemic, was taking portions of post mortem liver parenchyma [74,75].

In areas where systemic infections are prevalent, minimally invasive procedures with sampling of organs and tissues from cadavers can provide reliable data on specific causes of death [68].

Although collection of specimens from cadavers provided reliable data on specific causes of death and on detection of undocumented chains of transmission, only a full autopsy can investigate potential mechanisms of damage to organs or systems not readily accessible to biopsies, such as the central nervous system or cardiovascular system, leading to appropriate health-care strategies useful in the control of the disease [76].

Performing the histological and immunohistochemical investigations on the lung specimens collected during the autopsies of 8 subjects who died of SARS, the pathology of diffuse alveolar damage was described, providing the basis for therapeutic strategies [77].

Another important perspective that underlines the importance of autopsy is related to the development of vaccine candidates and new therapies for the prevention and treatment of lung disease caused by COVID-19 [4].

Moreover, about 7%–10% of the total amount of COVID-19 infected patients require admission to an ICU [10,14], and this percentage raises to about 35% among hospitalized patients with COVID-19 infection [15,16].

Despite the introduction of more modern diagnostic techniques and of intensive and invasive monitoring, the number of missed major diagnoses in ICU deceased has not essentially changed over the past 20 to 30 years [78]; autopsies revealed ante mortem diagnostic errors or ante mortem unrecognized diagnoses in about 30% of cases [79,80].

In a fascinating editorial published in 1970 in Chest, entitled “The Autopsy: Do We Still Need It?” [81], Edwards argued that despite the fact that the autopsy is the oldest method of medical investigation “... the current reports of autopsy findings serve to identify for current readers the nature of such conditions as a means of expanding the vistas of possible disease states”. Since 1970, as highlighted above, nothing has changed about the diagnostic role of the autopsy and—despite the improvement and reinforcement of any method of study in every field of medicine and science—there is no objective justification for eliminating it.

We firmly believe that the answer to Edward’s question in the 1970s is “yes, we still need the autopsy in 2020!” and we need it more nowadays, to answer a plethora of questions related to COVID-19 deaths.

As a scientific community, we are called on to face this global threat, and to defeat it with all available tools necessary, the new and the old ones, as the new and the old represent a proper union for continued progress in medicine.

This lesson, inherited from pioneers of medicine, should never be forgotten.

The time is now to shout out against this terrible lockdown of science: autopsy, autopsy, autopsy!

## Figures and Tables

**Table 1:** The main characteristics of the selected studies are summarized in the table. All studies were published in peer-reviewed journals indexed in PubMed in 2020.

Author	Number of Patients	Patients Who Died	Average Age (Years)	Comorbidities	Severe Complications	Tissues (Biopsy or Autopsy Samples)	Autopsy
Peng Y. D. et al. [8]	112	17	Data not available	Coronary heart disease (CHD) (100%), BMI >25 (88.24%)	Data not available	0	0
Tian S. et al. [9]	2	2	78.5	lung cancer (100%), hypertension (100%), diabetes (50%)	Respiratory failure, coma, heart failure	2 (biopsy)	0
Guan W. J. et al. [10]	1099	15	47	Hypertension (15%), diabetes (7.4%), CHD (2.5%), HCV (2.1%), chronic obstructive pulmonary disease (COPD) (1.1%), cancer (0.9%)	Septic shock, acute respiratory distress syndrome (ARDS), kidney failure	0	0
Xu Z. et al. [11]	1	1	50	Data not available	Respiratory failure, ARDS	1 (autoptic sample)	0
Kui K. et al. [12]	137	16	57	Hypertension (9.5%), diabetes (10.2%), CHD (7.3%), COPD (1.5%), cancer (1.5%)	Data not available	0	0
Wang W. et al. [13]	571	17	73	Hypertension (41.2%), diabetes (23.5%), CHD (17.6%), stroke (17.6%), COPD (11.7%), kidney failure (11.7%), Parkinson (11.7%), cancer (5.9%), cirrhosis (5.9%)	Data not available	0	0
Yang X. et al. [14]	201	32	59.7	CHD (9%), COPD (6%), diabetes (22%), cancer (3%), stroke (22%)	ARDS, kidney failure, heart failure, liver failure	0	0
Huang C. et al. [15]	41	6	49	Diabetes (20%), hypertension (15%), cardiovascular disease (CVD) (15%)	Respiratory distress syndrome (29%), RNAaemia (15%), acute cardiac injury (12%), secondary infection (10%)	0	0
Wang D. et al. [16]	138	6	56	Hypertension (31.2%), diabetes (10.1%), CVD (14.5%), Cancer (7.2%)	ARDS, arrhythmia, shock.	0	0
Chen N. et al. [17]	99	11	55.5	CVD and cerebrovascular diseases (40%), digestive system disease (11%), endocrine system disease (13%), cancer (1%), nervous system disease (1%), respiratory system disease (1%)	ARDS (17%), kidney failure (3%), respiratory failure (8%), Septic shock (4%)	0	0
Zhang et al. [18]	7	5	59	Not indicated	Acro-ischemia presentations including finger/toe cyanosis, skin bulla and dry gangrene (100%), definite disseminated intravascular coagulation (DIC) (4 (57%))	0	0
Korean Society of Infectious Diseases et al. [19]	54	54	75.5	CVD (59.3%); diabetes mellitus (DM) (29.6%); neurological disease (18.5%); lung disease (13.0%); malignancy (13.0%); psychologic disease (13.0%); renal disease (9.3%); hepatic disease (3.7%); kidney transplant recipient (1.9%)	Data not available	0	0
Yuan et al. [20]	27	10	60	Hypertension (19%); diabetes (22%); CVD (11%); tumor (4%); cerebral infarction (4%); chronic gastritis (4%)	ARDS (41%)	0	0
Guo et al. [21]	187	43	58.5	Hypertension (32.6%); CHD (11.2%); cardiomyopathy (8 (4.3%)); diabetes (15.0%); COPD (2.1); malignant neoplasm (7.0%); chronic kidney disease (CKD) (3.2%)	ARDS (24.6%), malignant arrhythmias (5.9%) including ventricular tachycardia/ventricular fibrillation, acute coagulopathy (34.1%), acute liver injury (15.4%) and acute kidney injury (14.6%)	0	0
Yang et al. [22]	25	5	60.2	Hypertension (15.4%); diabetes (7.7%); COPD (38.5%); CHD (30.8%)	Data not available	0	0
Chen et al. [23]	274	113	62	Hypertension (34%); diabetes (17%); CVD (8%); CHD (<1%); COPD (7%); malignancy (3%); hepatitis B (4%); cerebrovascular disease (1%); CKD (1%); gastrointestinal diseases (1%); metabolic arthritis (1%); autoimmune disease (1%)	ARDS (72%), type I respiratory failure (27%), acute cardiac injury (44%), heart failure (24%), hypoxic encephalopathy (9%), sepsis (65%), acidosis (12%), alkalosis (28%), acute kidney injury (11%), disseminated intravascular coagulation (8%), hyperkalemia (23%), shock (17%), acute liver injury (5%), gastrointestinal bleeding (<1%).	0	0
Zhuo et al. [24]	191	54	Data not available	Data not available	Sepsis (59%), respiratory failure (54%), ARDS (31%), heart failure (23%), septic shock (20%), coagulopathy (19%), acute cardiac injury (17%), acute kidney injury (15%), secondary infection (15%), hypoproteinemia (12%), acidosis (9%)	0	0
Deng et al. [25]	225	109	69	Hypertension (36.7%); lung disease (20.2%); diabetes (15.6%); heart disease (11.9%); malignancy (5.5%); others (28.4%)	ARDS (7.6%), acute cardiac injury (0.8%), acute kidney injury (<1%), shock (<1%), and disseminated intravascular coagulation (DIC) (<1%)	0	0
Rodriguez-Morales et al. [26]	2874	632	51.97	Hypertension (18.6%); CVD (14.4%); diabetes (11.9%); COPD (1.8%); malignancies (2.5%); chronic liver disease (CLD) (3.0%)	20.3% who required ICU: ARDS (32.8%), cardiac injury (13.0%), acute kidney injury (7.9%), shock (6.2%), Secondary infections (5.6%).	0	0
Guan et al. [27]	1590	50	48.9	Hypertension (16.7%); CVD (53.7%), cerebrovascular disease (1.9%), diabetes (8.2%), hepatitis B (1.8%), COPD (1.5%), CKD (1.3%), malignncy (1.1%)	Data not available	0	0
Bhatraju et al. [28]	24	12	64	Asthma (14%), CKD (21%), COPD (4%), tobacco smoker (22%), diabetes (58%),	Data not available	0	0
Zhang et al. [29]	28	8	65	Cancer (100%), diabetes 4(14.3%), COPD 1 (3.6%)	ARDS 8 (28.6%), septic shock 1 (3.6%), suspected pulmonary embolism 2 (7.1%), AMI 1 (3.6%)	0	0
Lescure et al. [30]	5	1	47	Hypertension 1 (20%), cancer 1 (20%), gout 1 (20%)	Data not available	0	0
Wu et al. [31]	201	44	51	Hypertension (19.4%) diabetes (10.9%) CVD (4.0%) liver disease (3.5%) nervous system disease (3.5%) chronic lung disease (2.5%) CKD (1.0%) endocrine system disease (1.0%) tumor (0.5%)	Data not available	0	0
Grasselli et al. [32]	1591	405	63	Hypertension (49%); CVD (21%); hypercholesterolemia (18%); DM (17); malignancy (8%); COPD (4%); CKD (3%); CLD (3%); other (20%)	Data not available	0	0
Bobin et al. [33]	10	4	68.4	Fracture (100%); hypertension (40%); diabetes (30%); COPD (10%); osteoporosis (30%); CHD (10%); cirrhosis (10%), alzheimer disease (10%); brain injury (10%)	Data not available	0	0
Chen et al. [34]	203	26	54	Hypertension (21.2%); diabetes (7.9%); CVD (7.9%); cerebrovascular disease (4.4%); malignancy (3,4%); CLD (3.9%); CKD (8 (3.9%)); COPD (8 (3.9%)); Tuberculosis (4 (2.0%)); HIV (2 (0.1%))	Cause of Death: ARDS (14%); ARDS with MOD (22%); sepsis/Shock (4%); heart failure (2%); myocardial infarction (6%); tumor (4%); intestinal bleeding (2%);	0	0
Li et al. [35]	25	25	73	Hypertension (64%); diabetes (40%); heart diseases (32%); kidney diseases (20%); cerebral infarction (16%); COPD (8%); malignant tumors (8%); acute pancreatitis (4%)	Respiratory failure (100%)	0	0
Cao et al. [36]	102	17	54	Hypertension (27.5%); diabetes (10.8%); cerebrovascular disease (5.9%); CVD (4.9%); respiratory diseases (9.8%); malignancy (3.9%); CKD (3.9%); CLD (2.0%);	Shock (9.8%); ARDS (19.6%); acute infection (16.7%); acute cardiac injury (14.7%); arrhythmia (17.6%); acute kidney injury (19.6%); acute liver injury (33.3%); lymphopenia (76.5%); Cause of Death: multiple organ dysfunction syndrome (MODS) (58.8%); ARDS (5.9%); cardiac arrest (23.5%); respiratory failure (11.8%)	0	0
Wang et al. [37]	339	65	71	Hypertension (40.8%), diabetes (16.0%), CVD (15.7%)	Lymphocytopenia (63.2%), bacterial infection (42.8%), liver enzyme abnormalities (28.7%), acute respiratory distress syndrome (21.0%)	0	0
Barton et al. [38]	2	2	77	Hypertension, splenectomy, cholelithiasis, osteoarthritis	Cause of Death: COVID-19 (ARDS—diffuse alveolar damage (DAD))	0	2
			42	Myotonic, muscular dystrophy	Complications of hepatic cirrhosis (aspiration acute bacterial bronchopneumonia)		
Huang et al. [39]	2	2	54.5	Transplantation (100%)	Nosocomial bacterial infection (100%); respiratory organ failure (100%); kidney organ failure (100%); heart organ failure (50%)	0	0
Ling et al. [40]	8	1	64.5	Data not available	Respiratory failure (75%); kidney failure (25%)	0	0
Cheng et al. [41]	701	113	63	Any comorbidity (42.6%); CKD (2.0%); COPD (1.9%); hypertension (33.4%); diabetes (14.3%); tumor (4.6%)	Acute kidney injury (5.1%)	0	0
Du et al. [42]	179	21	57.6	Hypertension (32.4%); CVD or cerebrovascular diseases (16.2%); diabetes (18.4%); chronic digestive disorders (11.7%); tuberculosis (4.5%); chronic hepatic or renal insufficiency (2.2%); Peripheral vascular disease (2.2%); malignancy (2.2%)	Data not available	0	0
Barrasa et al. [43]	48	14	63	Obesity (48%); arterial hypertension (44%); COPD (37%);	Hypoxemic respiratory failure (100%)	0	0
Lovell et al. [44]	101	75	82	Hypertension (54%); diabetes (36%); dementia (31%); cancer (25%); COPD (22%); renal failure (21%); congestive heart failure (18%); stroke / neurological disorder (12%); peripheral vascular disorder (4%); liver disease (2%);	Data not available	0	0
Wang et al. [45]	80	1	39	Hypertension (12.5%); diabetes (1.25%); CVD (2.5%); cerebrovascular disease (1.25%); COPD 1 (1.25%); renal disease (3.75%); liver disease (2.5%)	Data not available	0	0
Zhang et al. [46]	221	12	55	Hypertension (24.4%), diabetes (10.0%), CVD (10.0%), cerebrovascular disease (6.8%), COPD (2.7%), CKD (2.7%), CLD (3.2%), malignancy (4.1%), immunosuppression treatment (1.4%)	ARDS (21.7); arrhythmia (10.9); acute cardiac injury (7.7); shock (6.8); AKI (4.5)	0	0
Magro et al. [47]	5	2	54.6	Coronary artery disease, diabetes mellitus, heart failure, hepatitis C virus infection, end-stage renal disease, obesity (*n* = 2), and pre-diabetes,	Respiratory failure (*n* = 5); purpuric skin rash (*n* = 3)	3 cases skin biopsies	2 Cases, limited autopsy
Pereira et al. [48]	90	16	57	HTN (64%); diabetes (46%); CKD (63%); dialysis (6%); chronic lung disease (19%); HIV (1%); active cancer (3%); BMI >40 Kg/m^2^ (6%)	Data not available	0	0
Li et al. [49]	658	64	47	Cerebrovascular disease (8%); coronary heart disease (8.9%); heart failure (1.21%); diabetes (19.6%); hypertension (33.4%); digestive disorder (13.22%); COPD (2.88%); cancer (2.58%); CKD (2.73%); hepatitis (1.06%)	Ketosis (6.38%); acute liver injury (5.92%); septic shock (5.31%); ARDS (14.43%); diabetic ketoacidosis (DKA) (0.4%); acidosis (4.55%)	0	0
Yang et al. [50]	92	91	69.8	Hypertension (55.43%); heart disease (17.39%); cerebrovascular (10.8%); malignancy (4.34%); CLD (3.26%); CKD (2.17%); COPD (1%)	Cause of death: ARDS (79.34%), septic shock (7.6%), myocardial infarction (6.52%), heart failure (2.17%), MODS (2.17%)	0	0
Cai et al. [51]	7	3	61	COPD (28.5%); CVD (42.8%); interstitial lung disease (14.28%); hyperlipidemia (14.28%); Malignancy (100%)	Cause of death: respiratory failure 3	7 Biopsies	0
Karami et al. [52]	1	1	27	No underlying disease	Data not available	0	1
Tian et al. [53]	4	4	73	Chronic lymphocytic leukemia (CLL), cirrhosis, hypertension, diabetes, and renal transplantation	Data not available	4 (Autopsy samples)	0
Fabre et al. [54]	1	1	45	Obesity (BMI 40.4), hypertension	Pulmonary Embolism	0	0
Su et al. [55]	26	26	69	History of hypertension or diabetes or both (42.3%)	Data not available	26 (Kidney autopsy samples)	0
Edrada et al. [56]	2	2	42	Data not available	Data not available	0	0
Zhang et al. [57]	343	13	62	Diabetes (13.7%), hypertension (22.15%), CHD (5.5%), COPD (2.33%), cancer (2.62%), stroke history (2.33%), CLD (1.74%)	Data not available	0	0
